# Supplementary material for: Hydrostatic pressure drives sprouting angiogenesis via adherens junction remodelling and YAP signalling
Source: Commun Biol. 2024 Aug 3;7:940. doi: 10.1038/s42003-024-06604-9 (PMC11297954; doi:10.1038/s42003-024-06604-9)
Supplement: Supplementary file 2 — Supplementary Information file [file 42003_2024_6604_MOESM2_ESM.pdf]

Hydrostatic pressure drives sprouting angiogenesis via adherens junction remodelling and YAP signalling

Running title: Hydrostatic pressure on endothelia

Dunja Alexandra Al-Nuaimi[1], Dominic Rütsche[1], Asra Abukar[1], Paul Hiebert[2,#], Dominik Zanetti[3], Nikola Cesarovic[3,4], Volkmar Falk[3,4], Sabine Werner[2], Edoardo Mazza,[1,5,], Costanza Giampietro[1,5,*]*

[1] ETH Zürich, DMAVT, Experimental Continuum Mechanics, Zurich 8092, Switzerland

[2] ETH Zürich, Institute of Molecular Health Sciences, Department of Biology, 8093 Zurich, Switzerland.

[3] ETH Zürich, Department of Health Sciences and Technology, 8093, Zürich, Switzerland.

[4] Department of Cardiothoracic and Vascular Surgery, German Heart Center Berlin, Augustenburger Platz 1, 13353, Berlin, Germany.

[5] EMPA, Swiss Federal Laboratories for Materials Science and Technology, Experimental Continuum Mechanics, Dübendorf 8600, Switzerland

[#] present address: Centre for Biomedicine, Hull York Medical School, The University of Hull, Hull HU6 7RX, United Kingdom

[*] Corresponding authors:
E-mail: mazza@imes.mavt.ethz.ch; costanza.giampietro@empa.ch

Keywords: hydrostatic pressure, hemodynamics, endothelial cells, adherens junctions, YAP, vascular mechanobiology.

Supplemental Materials

Supplementary figures

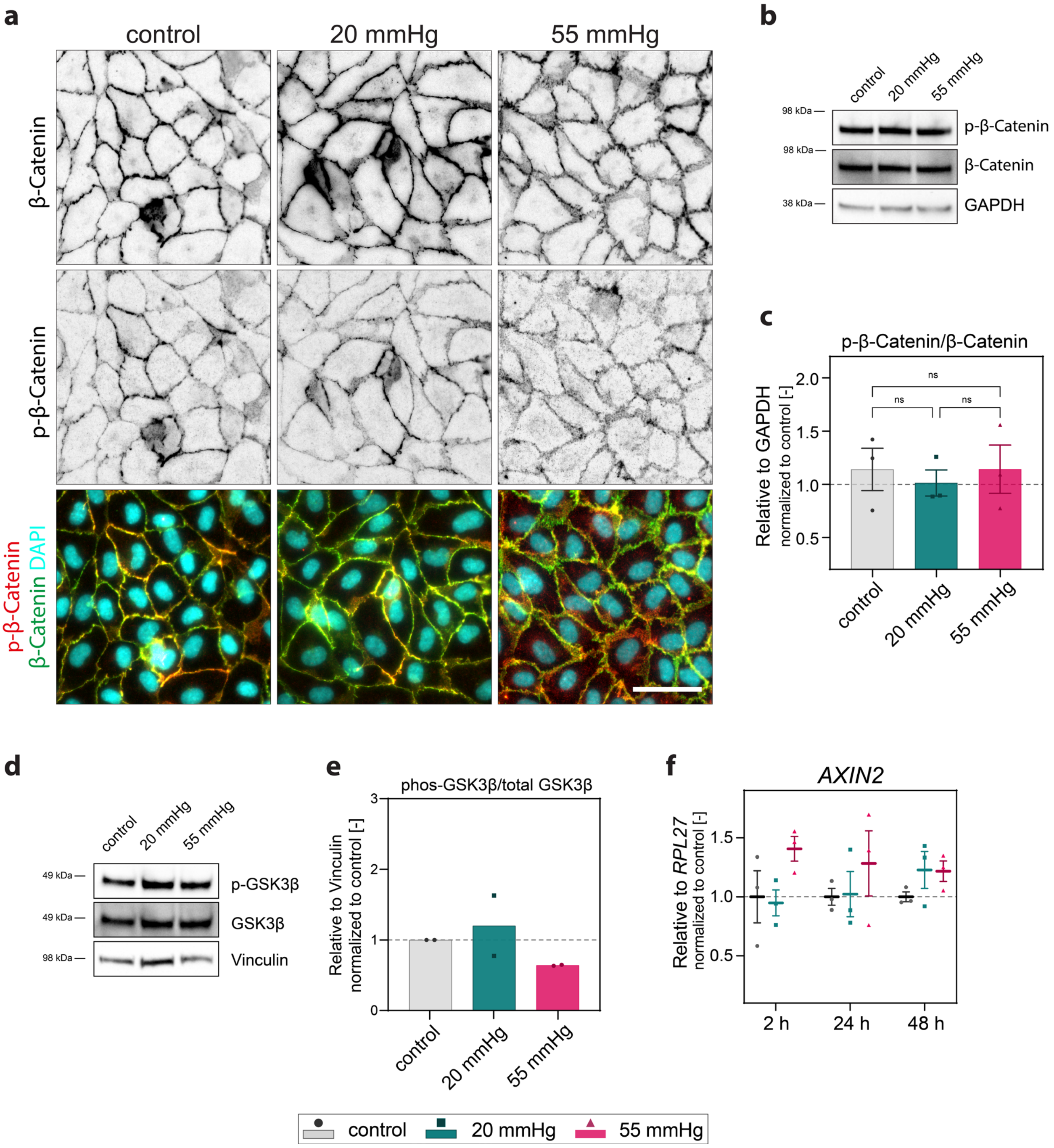

Figure S1. β-Catenin is not activated upon hydrostatic pressure stimulation:

a) β-Catenin and phospho-β-Catenin (Serine 552) staining of HUVECs after 24 h of hydrostatic pressure stimulation. Scale bar is 100 µm.

b) Western blot analysis of β-Catenin and p-β-Catenin (Serine 552) after exposure to pressure for 24 h, GAPDH is the loading control.

c) Densitometry of for ratio of p-β-Catenin (Serine 552) to β-Catenin protein abundance relative to GAPDH. N=3 independent experiments per group. Shown is mean ± s.e.m. ns = not significant; Kruskal-Wallis test.

d) Western blot analysis of GSK3β and phospho-GSK3β after exposure to pressure for 24 h, Vinculin is the loading control.

e) Densitometry of for ratio of p-GSK3β (Serine 9) to GSK3β protein abundance relative to Vinculin. N=2 independent experiments per group. Shown is mean ± s.d. ns = not significant, Friedman test.

f) RT-qPCR gene expression analysis of *AXIN2* relative to RPL27. N=3 independent experiments per group. Graphs show mean ± s.e.m. ns = non significant, for each timepoint one-way ANOVA with Dunnett's post-hoc test.

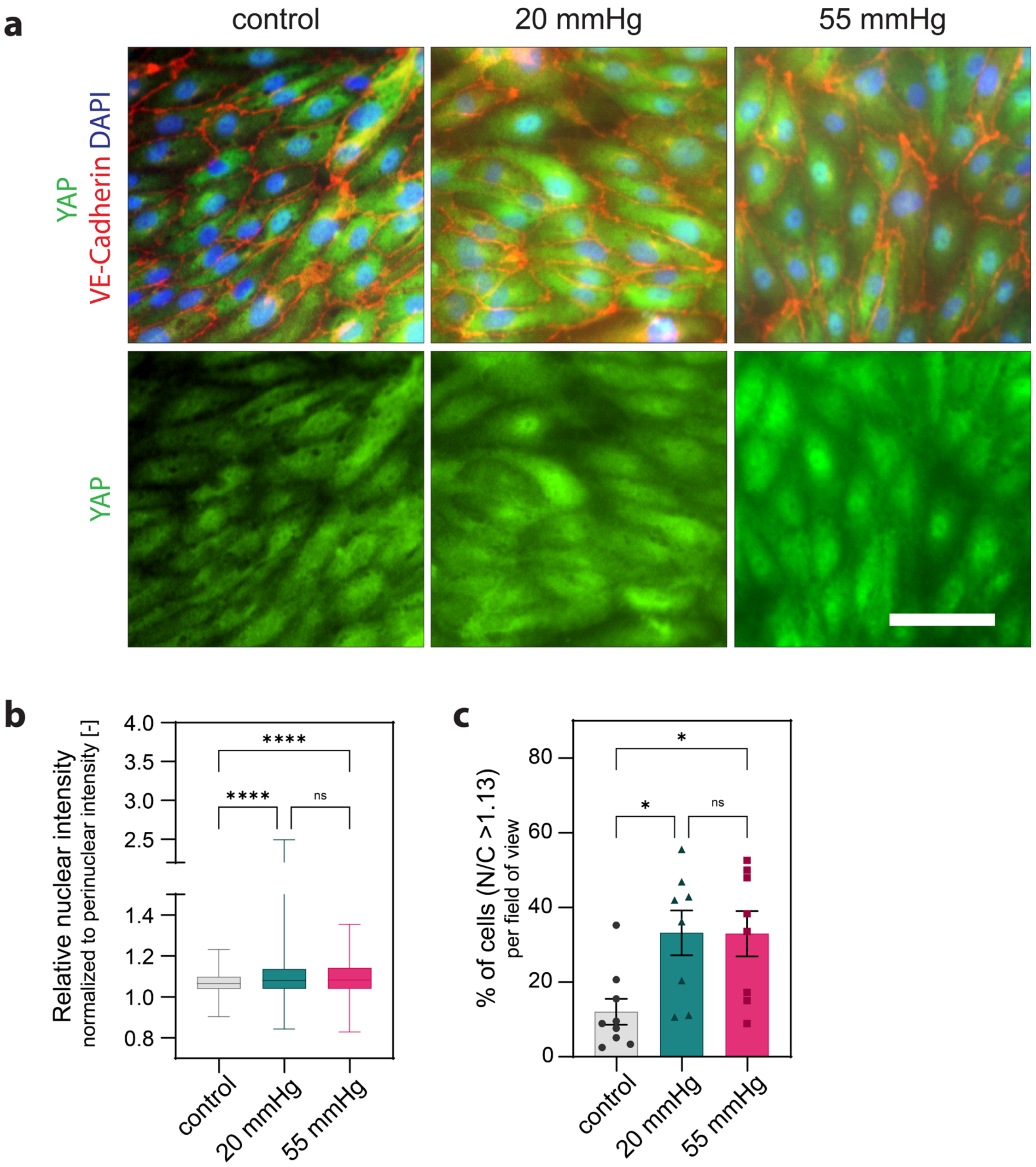

Figure S2. Hydrostatic pressure affects YAP intracellular localization of endothelial cells cultured on soft substrates:

a) Representative widefield z-projection images of HUVEC monolayers cultured on soft collagen hydrogels after 24 h under pressure showing YAP intracellular localization. VE-Cadherin (red), YAP (green), DAPI (blue).

b) Quantification of YAP nuclear localization after 24 h of stimulation (left). N=3 independent experiments per group, n≥2619 analysed nuclei pooled from ≥3 fields of view per condition and experiment.

c) Quantification of percentage of nuclei with a ratio of YAP nuclear/cytoplasmic signal > than 1.13. N=3 independent experiments, n≥ 8 fields of view.

Data information: b) Box plot shows median and 25th to 75th percentile, whiskers indicate min and max values. **** $p<0.0001$; Kruskal-Wallis test. c) Bar graphs show mean ± s.e.m. *$p<0.05$; one-way ANOVA with Tukey's post-hoc test. Scale bar is 50 μm.

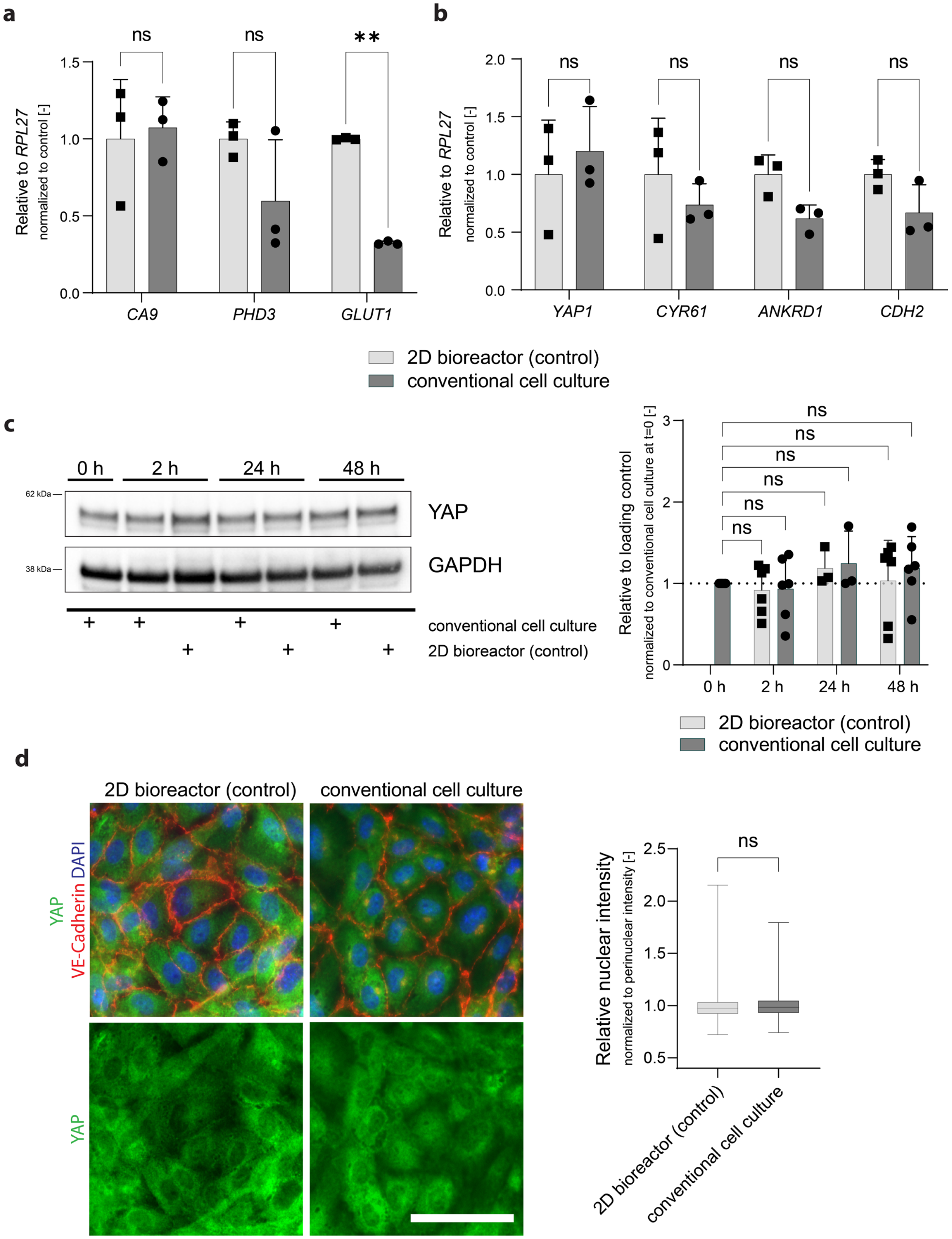

Figure S3. Cells cultured in 2D bioreactors do not show alterations in YAP expression and intracellular localization:

a) RT-qPCR of hypoxia-induced genes of endothelial monolayers cultured in the 2D bioreactor under 0 mmHg media column (control) compared to conventional cell culture (petri dish). N=3 independent experiments.

b) RT-qPCR of samples cultured in the 2D bioreactor under 0 mmHg media column in the bioreactor (control) compared to conventional cell culture (petri dish). N=3 independent experiments.

c) Western blot and densitometry of YAP protein expression over time of samples placed in 2D bioreactor (control) compared to samples maintained in conventional cell culture (petri dish). n≥3 independent experiments, shown is mean ± s.d., ns = not significant, two-way ANOVA (uncorrected Fisher's least significant difference).

d) Representative widefield z-projection images (left panel) and quantification (right panel) of monolayers cultured in 2D bioreactor (control) compared to samples maintained in conventional cell culture (petri dish) showing YAP intracellular localization. VE-Cadherin (red), YAP (green), DAPI (blue). N=3 independent experiments per group, n≥702 analysed nuclei pooled from ≥2 fields of view per condition and experiment.

Data information: d) Box plot shows median and 25th to 75th percentile, whiskers indicate min and max values. ns = non-significant; Kolmogorov-Smirnov test. Scale bar is 50 µm.

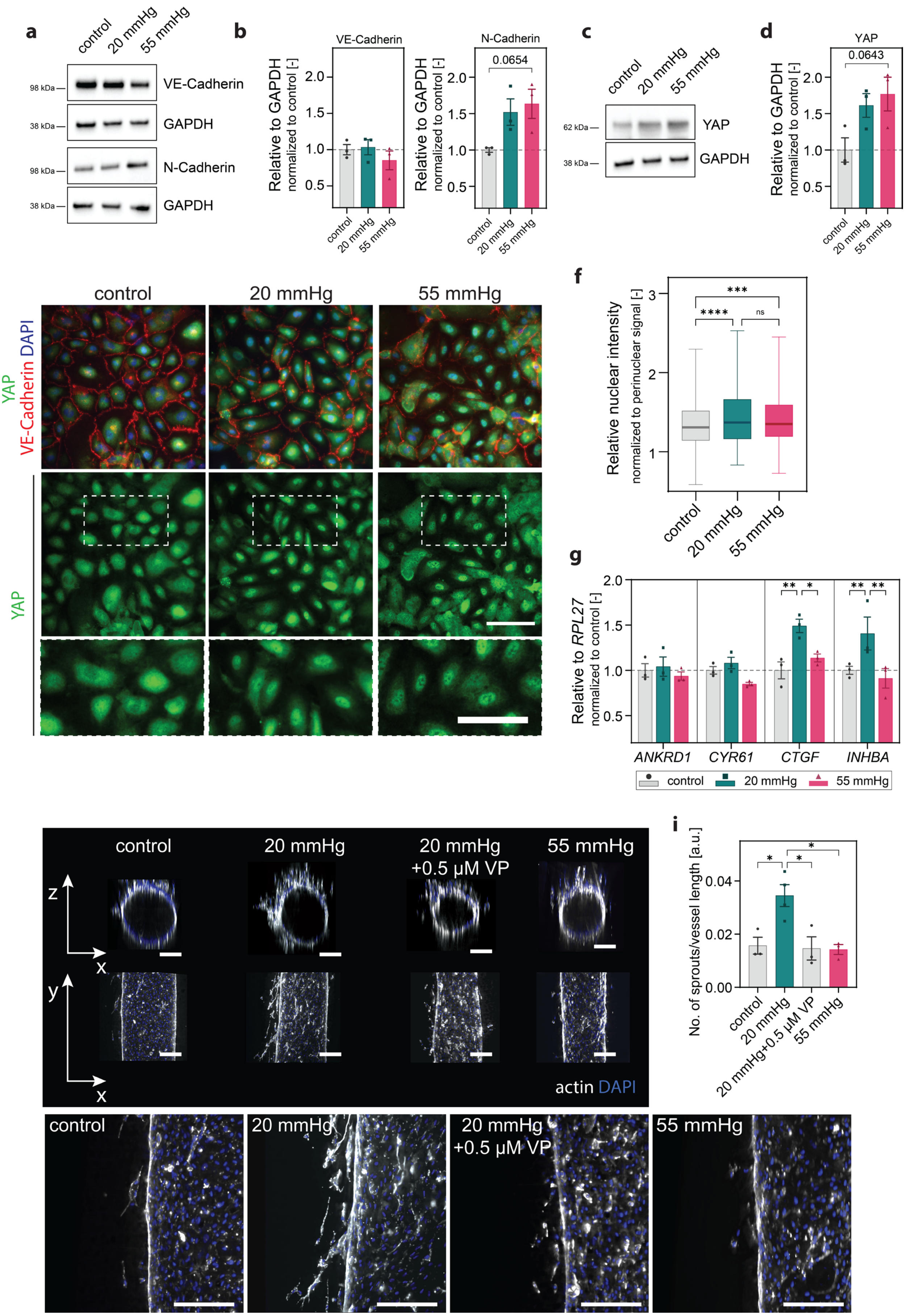
a
control
20 mmHg
55 mmHg
98 kDa
VE-Cadherin
38 kDa
GAPDH
98 kDa
N-Cadherin
38 kDa
GAPDH
b
VE-Cadherin
Relative to GAPDH
normalized to control [-]
2.0
1.5
1.0
0.5
control
20 mmHg
55 mmHg
N-Cadherin
0.0654
c
control
20 mmHg
55 mmHg
62 kDa
YAP
38 kDa
GAPDH
d
YAP
0.0643
Relative to GAPDH
normalized to control [-]
control
20 mmHg
55 mmHg
YAP VE-Cadherin DAPI
YAP
f
Relative nuclear intensity
normalized to perinuclear signal [-]
***
****
ns
3
2
1
control
20 mmHg
55 mmHg
g
Relative to RPL27
normalized to control [-]
2.0
1.5
1.0
0.5
**
*
**
**
ANKRD1
CYR61
CTGF
INHBA
control
20 mmHg
55 mmHg
i
control
20 mmHg
20 mmHg
+0.5 µM VP
55 mmHg
z
x
y
x
actin DAPI
No. of sprouts/vessel length [a.u.]
0.04
0.02
0.00
*
*
*
control
20 mmHg
20 mmHg+0.5 µM VP
55 mmHg
control
20 mmHg
20 mmHg
+0.5 µM VP
55 mmHg

Figure S4. Hydrostatic pressure induced YAP activation and sprouting angiogenesis in HAoECs:

a) Western blot analysis of VE-Cadherin and N-Cadherin in HAoECs after exposure to pressure for 24 h, GAPDH is the loading control.

b) Densitometry of N-Cadherin and VE-Cadherin protein abundance relative to GAPDH. N=3 independent experiments per group.

c) Western blot analysis of YAP in HAoECs after exposure to pressure for 24 h, GAPDH is the loading control.

d) Densitometry of YAP protein abundance relative to GAPDH. N=3 independent experiments per group.

e) Representative widefield z-projection images of monolayers after 24 h under pressure showing YAP intracellular localization. VE-Cadherin (red), YAP (green), DAPI (blue).

f) Quantification of YAP nuclear localization after 24 h of stimulation. N=3 independent experiments per group, n≥ 648 analysed nuclei pooled from ≥3 fields of view per condition and experiment.

g) RT-qPCR gene expression analysis of the YAP target genes *CTGF*, *CYR61*, *INHBA* and *ANKRD1* relative to *RPL27.* N=3 independent experiments per group.

h) Deconvoluted representative widefield projection images of 3D HAoECs organotypic models after 48 h under pressure stimulation with and without 0.5 µM VP. Actin (gray), DAPI (blue). Representative magnifications show angiogenic sprouts visualized by altered brightness, contrast, and projected planes of control, 20 mmHg, 20 mmHg + 0.5 µM VP, and 55 mmHg conditions.

i) Quantification of sprouts in 3D HAoECs organotypic models after 48 h under pressure. N≥3 independent experiments per group.

Data information: a, b) Bar graphs show mean ± s.e.m. one-way ANOVA with Tukey's post-hoc test. f) Box plot shows median and 25th to 75th percentile, whiskers indicate min and max values. ***p<0.001, ****p<0.0001; Kruskal-Wallis test with Dunn's post hoc test. g) Graph shows mean ± s.e.m. **p<0.01 two-way ANOVA with Tukey's post-hoc test, for each gene. i) Bar Graph shows mean ± s.e.m. ***p<0.001, ****p<0.0001; one-way ANOVA with Tukey's post-hoc test. Scale bars: 100 µm.

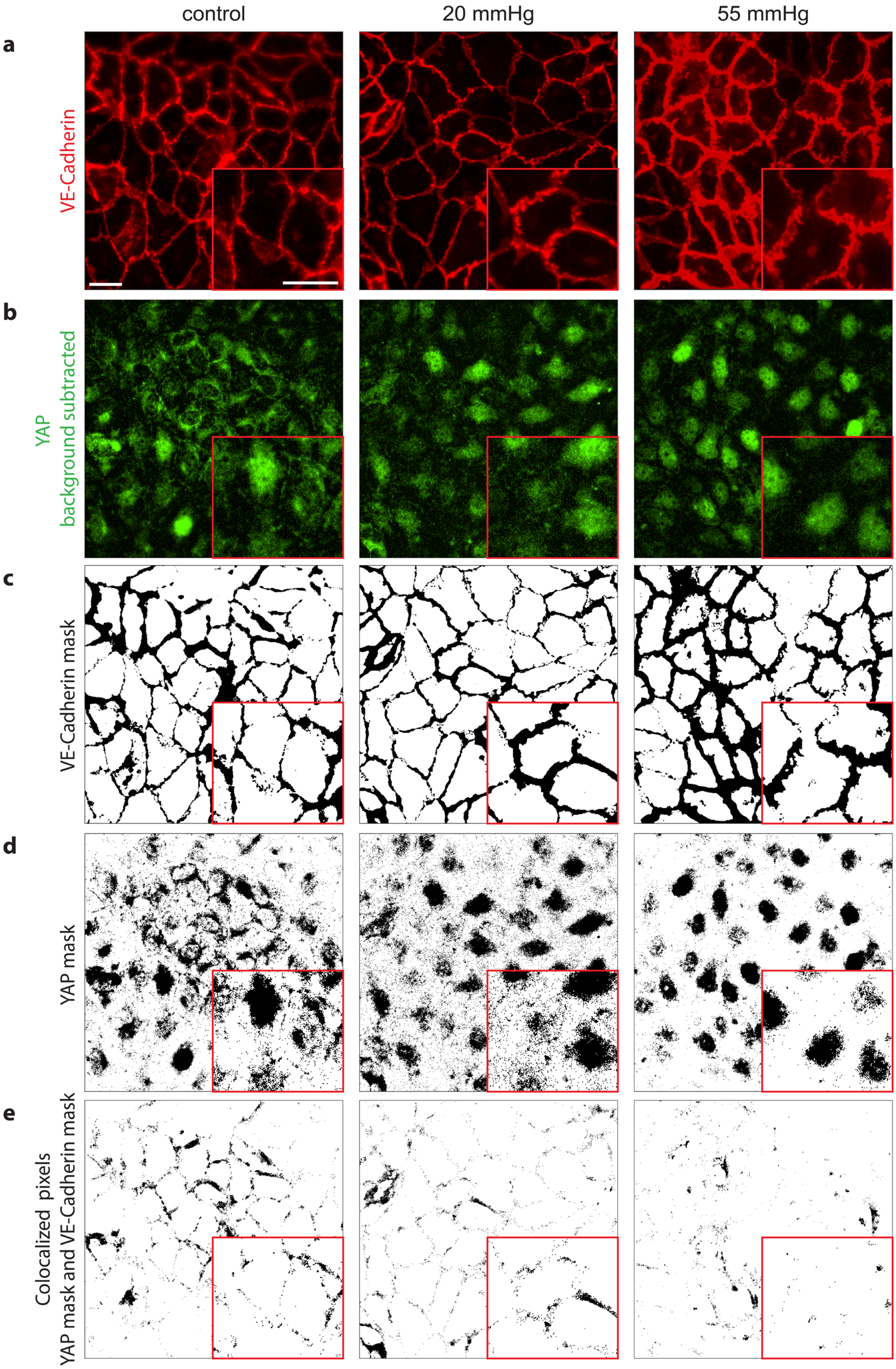
control
20 mmHg
55 mmHg
a
VE-Cadherin
b
YAP
background subtracted
c
VE-Cadherin mask
d
YAP mask
e
Colocalized pixels
YAP mask and VE-Cadherin mask

Figure S5. Pipeline of the analysis of YAP junctional localization:

Representative z-projections of VE-cadherin (a, red) and YAP (b, green) signals. Binarized VE-cadherin signal (c, mask), binarized YAP signal (d, mask). Scale bars are 50 µm.

e) Colocalized pixels of c and d images.

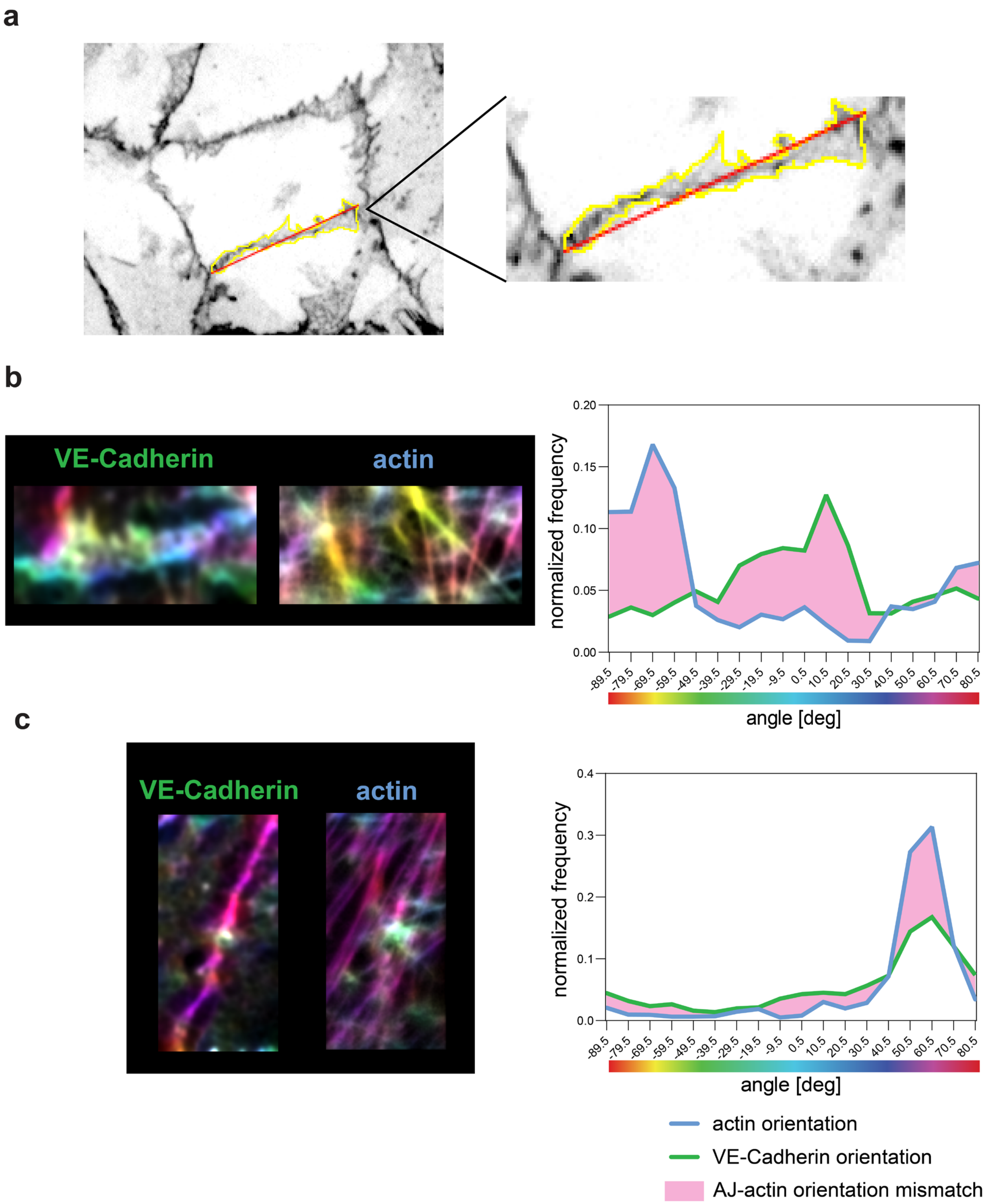

Figure S6. Pipeline of the analysis of AJ-actin orientation:

a) Junction morphological analysis from z-stack projection images of VE-Cadherin staining. yellow: one “junction” as selected from vertex to vertex. red: Feret’s diameter (longest distance between any two points).

b and c) Representative analysis of orientation mismatch between a single adherens junction and the local actin cytoskeleton from the 55 mmHg (b) and control condition (c). Left: Immunofluorescence z-projection images coloured according to orientation. Right: Corresponding histogram line graphs of actin and VE-Cadherin orientation.

Figure S7. Uncropped western blot images for all figures.

Figure 5c

VE-Cadherin

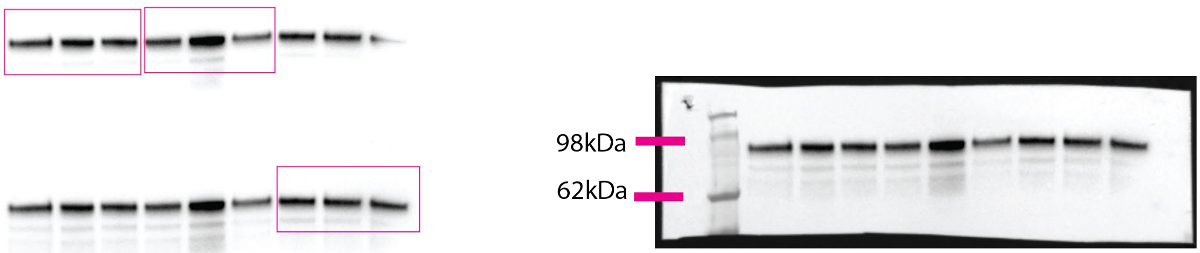

N-Cadherin

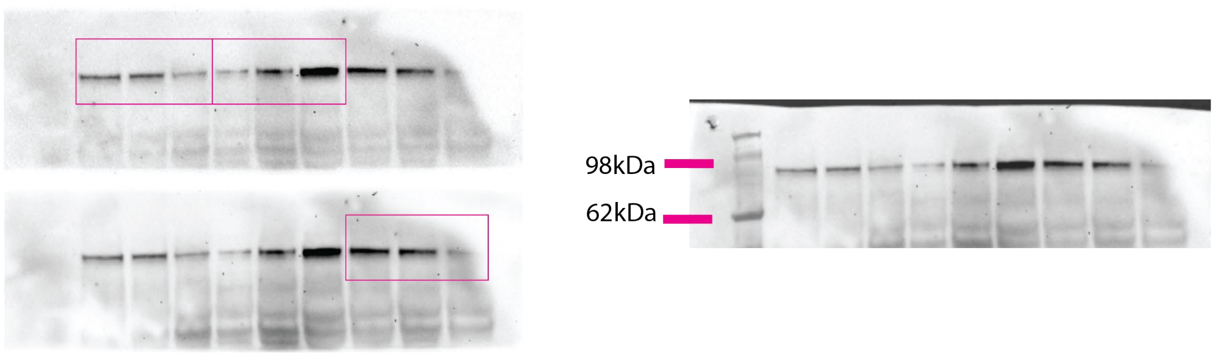

GAPDH

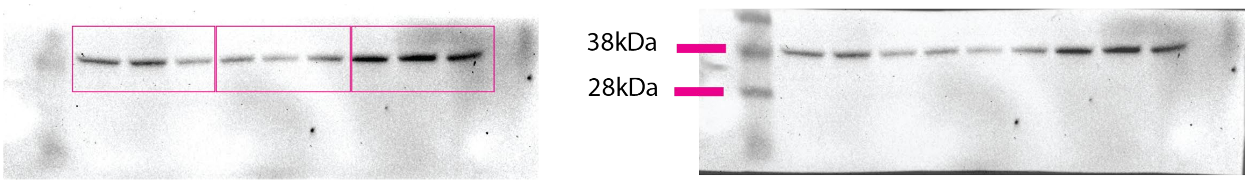

Figure 6d

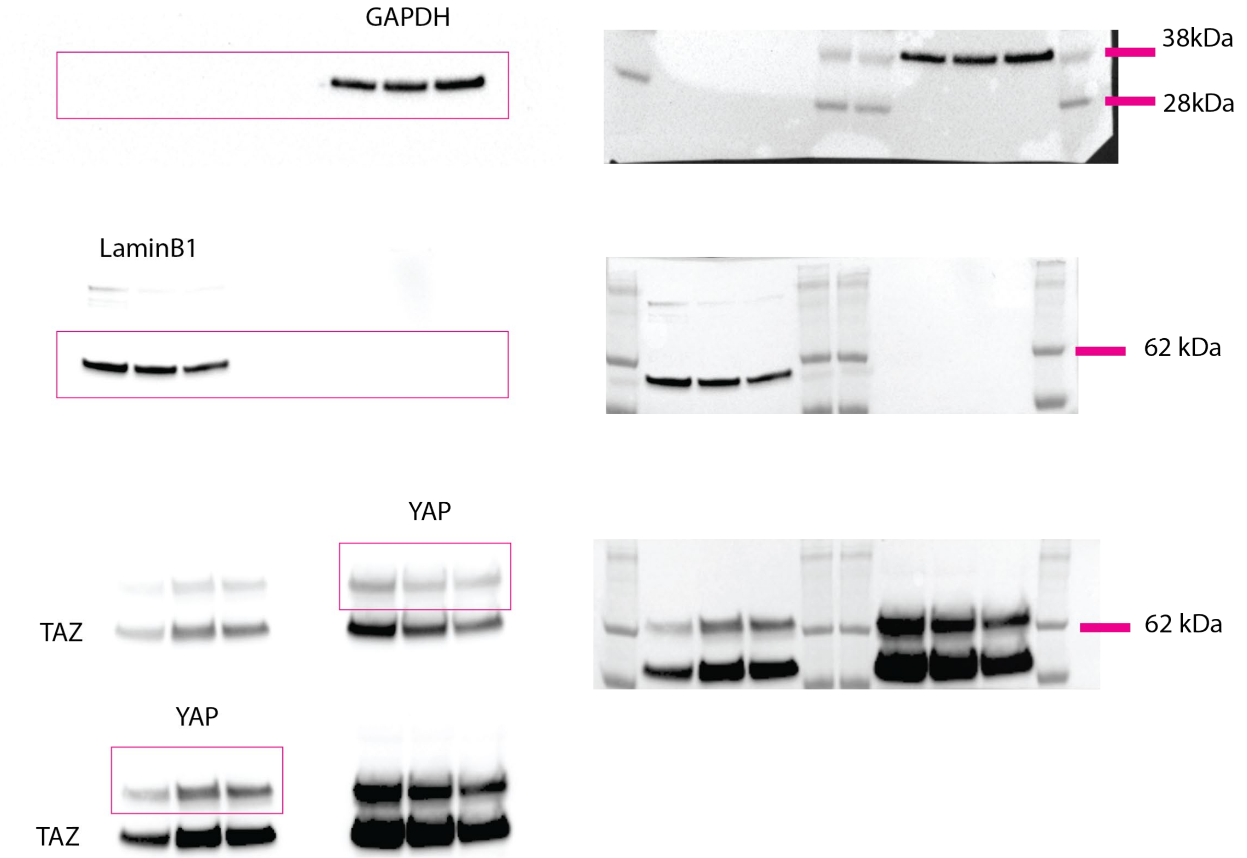

Figure 6f

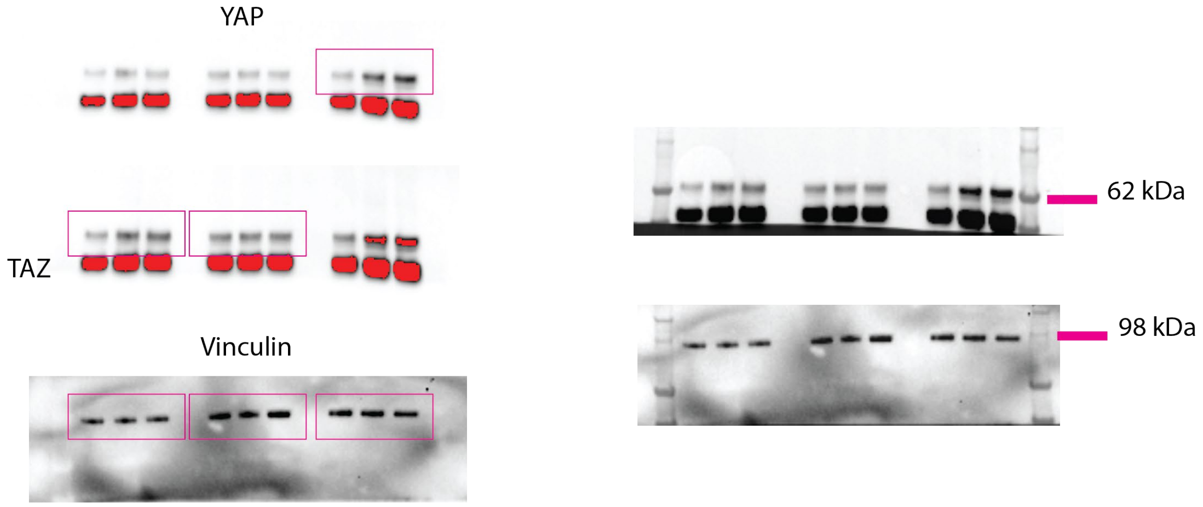

Figure S1b

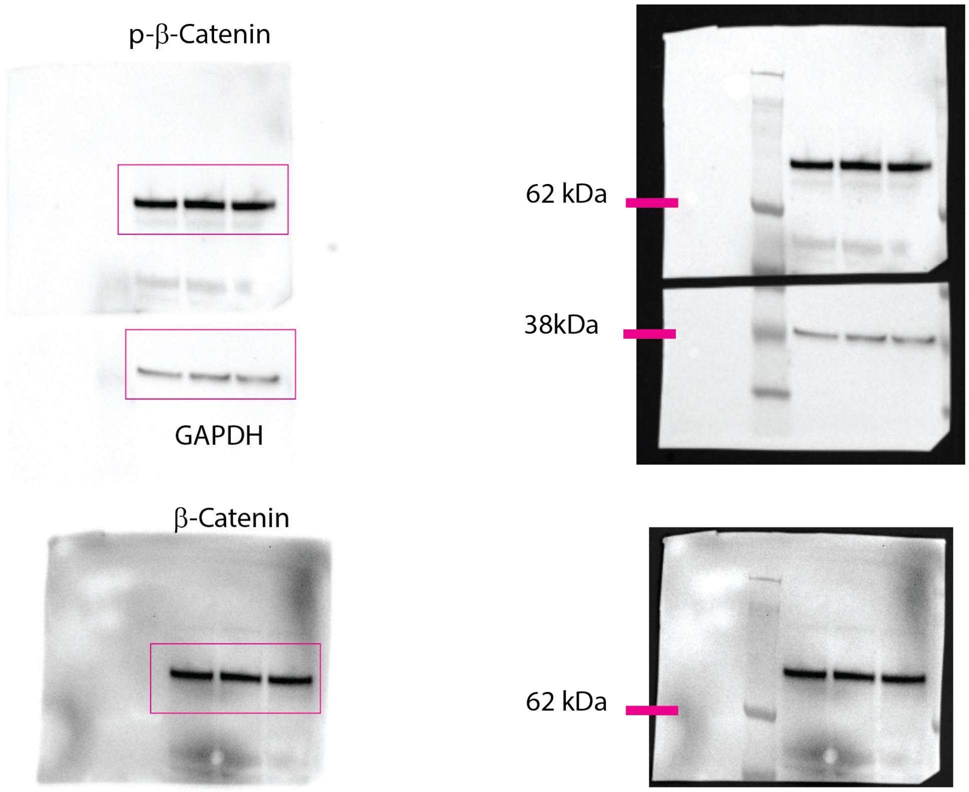

Figure S1d

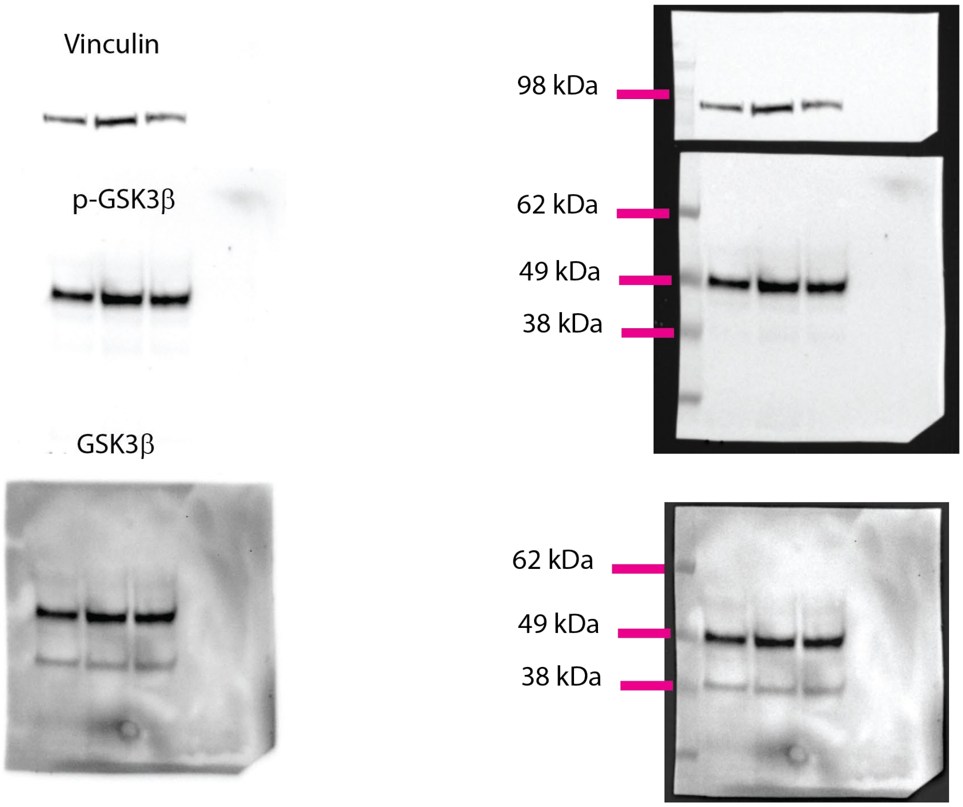

Figure S3c

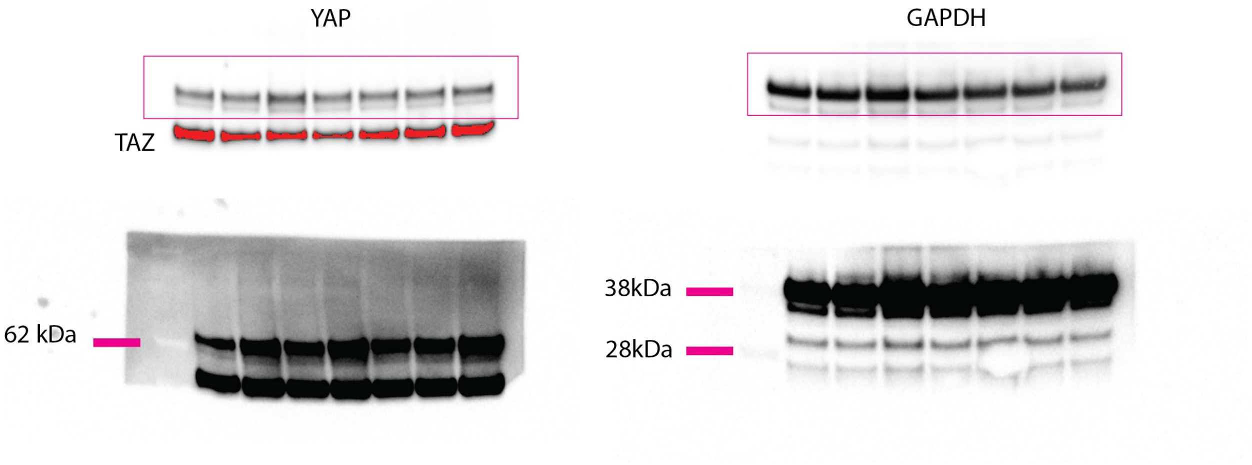

Figure S4a, c

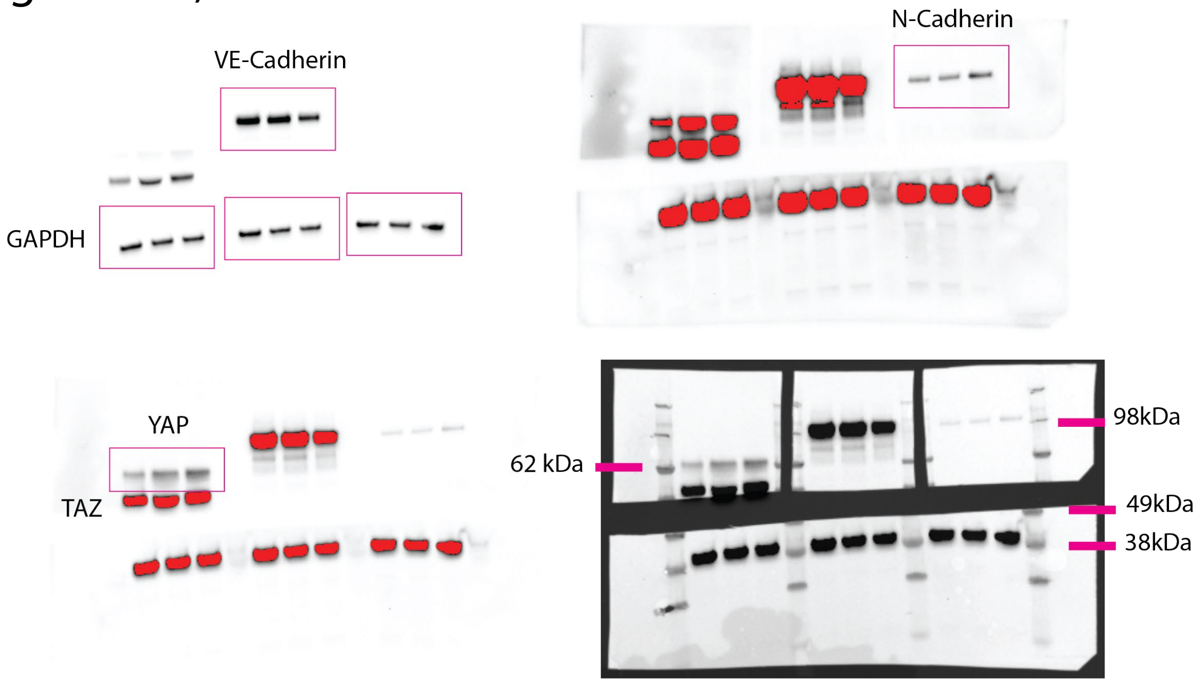

Gene	*Primer sequence (5'-3') forward; reverse (5'-3')*
ACTA2	AAAAGACAGCTACGTGGGTGA;<br>GCCATGTTCTATCGGGTACTTC
ANGPT2	ATTCAGCGACGTGAGGATGGCA;<br>GCACATAGCGTTGCTGATTAGTC
ANKRD1	CGACTCCTGATTATGTATGGCGC;<br>GCTTTGGTTCCATTCTGCCAGTG
AXIN2	CAAACTTTCGCCAACCGTGGTTG;<br>GGTGCAAAGACATAGCCAGAACC
CA9	GTGCCTATGAGCAGTTGCTGTC;<br>AAGTAGCGGCTGAAGTCAGAGG
CTGF	CTTGCGAAGCTGACCTGGAAGA;<br>CCGTCGGTACATACTCCACAGA
CYR61	GGAAAAGGCAGCTCACTGAAGC;<br>GGAGATACCAGTTCCACAGGTC
FGF2	AGCGGCTGTACTGCAAAAACGG;<br>CCTTTGATAGACACAACTCCTCTC
GLUT1	TTGCAGGCTTCTCCAACTGGAC;<br>CAGAACCAGGAGCACAGTGAAG
N-CADHERIN (CDH2)	CCTCCAGAGTTTACTGCCATGAC;<br>GTAGGATCTCCGCCACTGATTC
PHD3	GAACAGGTTATGTTCGCCACGTG;<br>CCCTCTGGAAATATCCGCAGGA
RPL27	TCACCTAATGCCCACAAGGTA;<br>CCACTTGTTCTTGCCTGTCTT
SLUG	AGATGCATATTCGGACCCAC;<br>CCTCATGTTTGTGCAGGAGA
SMAD2	CGTCCATCTTGCCATTCACG;<br>CTCAAGCTCATCTAATCGTCCTG
VE-CADHERIN (CDH5)	GAAGCCTCTGATTGGCACAGTG;<br>TTTTGTGACTCGGAAGAACTGGC
VEGFA	TTGCCTTGCTGCTCTACCTCCA;<br>GATGGCAGTAGCTGCGCTGATA

VEGFR2	GGAACCTCACTATCCGCAGAGT;<br>CCAAGTTCGTCTTTTCCTGGGC
YAP	TGTCCCAGATGAACGTCACAGC;<br>TGGTGGCTGTTTCACTGGAGCA
ZEB2	ATAAGGGAGGGTGGAGTGGA;<br>CGCGTTCCTCCAGTTTTCTT
INHBA	CCTCGGAGATCATCACGTTT;<br>CCCTTTAAGCCCACTTCCTC

Table S1: Primer sequences for RT-qPCR (obtained from OriGene (OriGeneTechnologies))

Product	*Supplier, Reference N^o.*	*Dilution immuno-fluorescence*	*Dilution Western blot*
Alexa Fluor™ 488 Phalloidin	Thermo Fisher Scientific, A12379	1:500	-
chicken anti-mouse Alexa Fluor™ 647	Thermo Fisher Scientific, A21463	1:250	-
chicken anti-rabbit Alexa Fluor™ 647	Thermo Fisher Scientific, A21443	1:250	-
DAPI	Thermo Fisher Scientific, 62248	1:1000	-
donkey anti-mouse Alexa Fluor™ 488	Thermo Fisher Scientific, A21202	1:250	-
donkey anti-mouse Alexa Fluor™ 555	Thermo Fisher Scientific, A31570	1:250	-
donkey anti-rabbit Alexa Fluor™ 488	Thermo Fisher Scientific, A21206	1:250	-
donkey anti-rabbit Alexa Fluor™ 555	Thermo Fisher Scientific, A32794	1:1000	-
EZ-Link™ NHS-LC-LC-Biotin	Thermo Fisher Scientific 21343	0.57 mg/ml	
GAPDH	Santa Cruz, sc-47724	-	1:800
GSK3β	Cell Signaling Technology, 9832	-	1:500
IsolectinB4	Vector Laboratories, B-1205	1:100	-
KI-67	BD Biosciences, 550609	1:250	-
Lamin B1	abcam, ab65986	-	1:1000
N-Cadherin	BD Biosciences, 610921	1:500	1:2500
p-GSK3β (S9)	Cell Signaling Technology, 93235	-	1:500
p-β-Catenin (S552)	Cell Signaling Technology, 5651	1:100	1:1000

Streptavidin-488	Thermo Fisher Scientific, S11223	1:500	-
VE-Cadherin	Cell Signaling Technology, 2500	1:500	1:1000
Vinculin	Sigma-Aldrich, V4505	-	1:1000
YAP	Santa Cruz, sc-101199	1:250	1:1000
β-Catenin	BD Biosciences, 610154	1:200	1:1000

Table S2. List of antibodies, chemicals, and fluorescent dyes.
