# Supplementary material for: Hydrostatic pressure drives sprouting angiogenesis via adherens junction remodelling and YAP signalling
Source: Commun Biol. 2024 Aug 3;7:940. doi: 10.1038/s42003-024-06604-9 (PMC11297954; doi:10.1038/s42003-024-06604-9)
Supplement: Supplementary file 3 — Description of Additional Supplementary Materials [file 42003_2024_6604_MOESM3_ESM.pdf]

Description of Additional Supplementary Files

File name: Supplementary Data 1

Description: excel file with raw numerical source data for graphs and charts of the main figures

File name: Supplementary Data 2

Description: excel file with raw numerical source data for graphs and charts of the Supplementary figures.
